# Associations between steatotic liver disease subtypes and incident atrial fibrillation in young adults: a nationwide cohort study

**DOI:** 10.1186/s12933-025-02905-3

**Published:** 2025-08-25

**Authors:** Jeayeon Park, Goh Eun Chung, Su Jong Yu, Yoon Jun Kim, Jung-Hwan Yoon, Kyungdo Han, Eun Ju Cho

**Affiliations:** 1 Department of Internal Medicine and Liver Research Institute, Seoul National University College of Medicine, Seoul, Republic of Korea; 2 Department of Internal Medicine and Healthcare Research Institute, Seoul National University Hospital Healthcare System Gangnam Center, Seoul, Republic of Korea; 3 Department of Statistics and Actuarial Science, Soongsil University, Seoul, Republic of Korea

## Abstract

**Background/aim:**

Metabolic dysfunction is emerging as a significant risk factor for atrial fibrillation (AF) and serves as a foundational component of both metabolic dysfunction-associated steatotic liver disease (MASLD) and MASLD with moderate alcohol intake (MetALD). As the prevalence of steatotic liver disease (SLD) rises among young adults, clarifying its association with AF in this population has become a clinical priority. Accordingly, we aimed to investigate the link between different SLD subtypes and the risk of incident AF in young adults.

**Methods:**

In this nationwide cohort study, we analyzed data from the Korean National Health Insurance Service and included individuals aged 20–39 years who underwent health screening examinations between 2009 and 2012. The participants were categorized into either the non-SLD group or the SLD group, which was defined by a fatty liver index ≥ 30. SLD was further subclassified into MASLD, MetALD, and alcohol-associated liver disease (ALD) for analysis. The risk of incident AF was evaluated using Cox proportional hazards models.

**Results:**

A total of 6,375,710 young adults (mean age 30.9 years; 59.4% male) were included, with a median follow-up period of 10.6 years. The prevalence of SLD was 27.8%, which included cases of MASLD (81.7%), MetALD (13.5%), and ALD (4.7%). The risk of incident AF was significantly elevated in individuals with SLD, with progressive increases across MASLD, MetALD, and ALD subtypes. Compared with those of the non-SLD group, the adjusted hazard ratios for AF were 1.09 (95% confidence interval [CI], 1.05–1.31) in the MASLD group, 1.29 (95% CI, 1.22–1.36) in the MetALD group, and 1.52 (95% CI, 1.41–1.65) in the ALD group.

**Conclusion:**

SLD is associated with new-onset AF, with a progressively increased risk across MASLD, MetALD, and ALD subtypes in young adults. Given the modifiable nature of these risks, early interventions are essential to prevent long-term cardiovascular complications and reduce the future disease burden.

**Supplementary Information:**

The online version contains supplementary material available at 10.1186/s12933-025-02905-3.

## Introduction

Steatotic liver disease (SLD**)**, characterized by more than 5% of hepatocytes containing fat, is an umbrella term with subtypes including metabolic dysfunction-associated steatotic liver disease (MASLD), metabolic dysfunction and alcohol-related liver disease (MetALD), alcohol-associated liver disease (ALD), SLD with a specific etiology, and cryptogenic SLD [1, 2]. Metabolic dysfunction, a key component of MASLD and MetALD, may play a role in the development of cardiovascular disease (CVD) [3].

Atrial fibrillation (AF) is one of the most common symptomatic arrhythmias worldwide and can occur even in the absence of underlying heart disease [4, 5]. With the identification of various risk factors for AF, recent focus has shifted toward modifiable contributors to prevent its onset and progression [6]. Among these factors, cardiometabolic risk factors and alcohol consumption are well recognized [7–9]. Clusters of cardiometabolic abnormalities are strongly associated with the development and pathogenesis of AF [7, 10, 11]. Furthermore, several meta-analyses have demonstrated that even moderate, habitual alcohol consumption increases the risk of AF in a dose-dependent manner [12–14]. In this context, MASLD, MetALD, and ALD—conditions characterized by underlying cardiometabolic dysfunction and/or alcohol use—may be linked to an increased risk of AF.

Although the association between MASLD and AF has been reported in the general population [15], limited evidence exists regarding the incidence of AF, especially in young adults with coexisting SLD. As the global prevalence of SLD continues to increase, it has emerged as a major cause of chronic liver disease in both children and young adults [16]. This trend is particularly concerning, as the longer life expectancy of young adults may lead to prolonged exposure to MASLD and, consequently, a greater cumulative risk of developing long-term complications. A previous study reported an association between nonalcoholic fatty liver disease (NAFLD) and incident AF in young adults; however, this study used an exclusionary definition of NAFLD [17]. With the recent international consensus shifting the nomenclature to SLD, there is a critical need to understand how the newly defined and clinically distinct SLD subtypes contribute to the risk of AF. Therefore, the primary aim of the present study was to investigate the incidence of new-onset AF in young adults specifically according to these new, clinically distinct SLD subtypes.

## Patients and methods

### Study population

This study utilized data from the National Health Insurance Service (NHIS) database of Korea, which is administered by the Korean government and encompasses nearly 97% of the population. This database contains comprehensive health records, including demographic information, anthropometric measurements (height, weight, waist circumference [WC], systolic and diastolic blood pressures), laboratory test results (fasting glucose, total cholesterol, triglycerides, high-density lipoprotein [HDL] and low-density lipoprotein [LDL] cholesterol, aspartate aminotransferase, alanine aminotransferase, gamma-glutamyl transferase [GGT], and creatinine), lifestyle factors (smoking, alcohol consumption, and regular exercise), medical diagnoses coded according to the 10th revision of the International Classification of Diseases (ICD-10), and treatment details [18]. The exact diagnostic codes used for this study are described in Supplementary Table 1. Outcomes were primarily ascertained through claim data submitted by healthcare providers for reimbursement. The NHIS standardizes coding and claims processing to ensure data consistency. Despite near-universal healthcare coverage in South Korea, certain limitations such as loss to follow-up and censoring may affect data completeness.

The study population included individuals aged 20–39 years who underwent health screening examinations between 2009 and 2012, with follow-up continuing until 2021. To focus on the main etiological factors (i.e., cardiometabolic risk factors and alcohol) of the MASLD criteria, we excluded individuals with concomitant liver disease or cryptogenic SLD with a distinct etiological background. Patients who met any of the following criteria were excluded: (i) a prior diagnosis of AF; (ii) a history of liver transplantation (ICD-code, Z94.4, T86.4, V013); (iii) a prior diagnosis of hepatocellular carcinoma (ICD-code, C22 and V193); (iv) preexisting concomitant liver disease, a current diagnosis of cryptogenic SLD, specific etiology of SLD (ICD-code, Supplementary Table 1); (v) incomplete clinical data; and (vi) a new diagnosis related to the primary outcomes or death within one year of the health screening (Fig. 2).

**Fig. 1:**
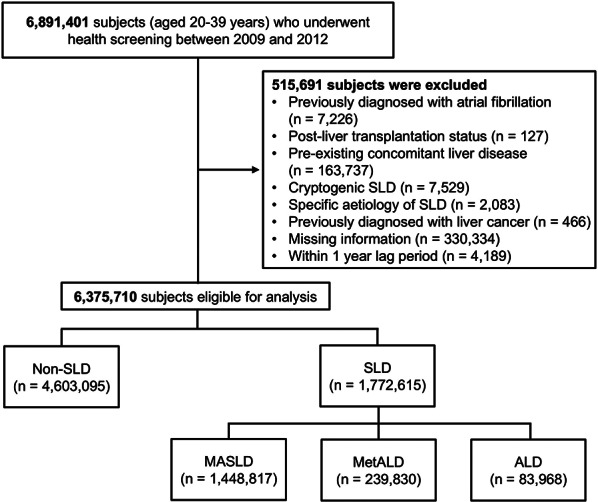
Flow diagram of the study population. ALD, alcohol-associated liver disease; MASLD, metabolic dysfunction-associated steatotic liver disease; MetALD, metabolic dysfunction and alcohol-related liver disease; SLD, steatotic liver disease

This study conformed to the ethical guidelines of the World Medical Association Declaration of Helsinki. This study was approved by the Institutional Review Board of Seoul National University Hospital (IRB No. H-2401-130-1505). The requirement to obtain informed consent from patients was waived by the IRB because of the retrospective nature of this study.

## Assessments and definitions

Given the retrospective nature of the study, the collection of data from liver biopsies was unfeasible; instead, the widely used and noninvasive fatty liver index (FLI) was utilized to define the presence of SLD [19, 20]. It was previously validated in a Korean population for the detection of ultrasound-diagnosed fatty Liver with areas under the receiver operating characteristic curves of 0.79–0.87 [21, 22]. The FLI was calculated via the following equation on the basis of body mass index (BMI), WC, triglyceride, and GGT data. In this study, an FLI score ≥ 30 was used to define the presence of SLD.

$$\begin{aligned}\text{FLI} & = \left[\left(e^{ \begin{array}{l} 0.953 \times \text{ln triglyceride} + 0.139 \times \text{BMI} + 0.718\\ \times \text{ln GGT}\\ + 0.053 \times \text{WC} - 15.745 \end{array} } \right) / \right. \\ & \qquad \left. \left(1 + e^{ \begin{array}{l}0.953 \times \text{ln triglyceride} + 0.139\\ \times \text{BMI} + 0.718 \times \text{ln GGT} \\ + 0.053 \times \text{WC} - 15.745 \end{array} } \right ) \vphantom{e^{ \begin{array}{l} sjb\\sdnj \end{array}} } \right ] \times 100 \end{aligned}$$

MASLD was defined as the presence of SLD without any other identifiable causes of steatosis in combination with one or more of the following cardiometabolic risks [23]: (i) a BMI ≥ 23 kg/m² or a WC ≥ 90 cm for men and ≥ 85 cm for women [24]; (ii) a blood pressure ≥ 130/85 mmHg or treatment with antihypertensive medication; (iii) a fasting plasma triglyceride level ≥ 150 mg/dL or the use of lipid-lowering therapy; (iv) a plasma HDL cholesterol level < 40 mg/dL for men and < 50 mg/dL for women or the use of cholesterol-lowering treatment; and (v) a fasting glucose level ≥ 100 mg/dL, a diagnosis of type 2 diabetes mellitus (T2DM), or treatment for T2DM. MetALD was defined as MASLD with concurrent alcohol intake of 30–60 g/day for males and 20–50 g/day for females. ALD was defined as the presence of SLD in individuals whose alcohol consumption exceeded 50 g/day for women and 60 g/day for men or a diagnosis of alcohol abuse/alcohol-related liver disease (ICD-code, Supplementary Table 1).

## Study outcomes

The primary outcome was the incidence of new-onset AF. New-onset AF was defined as at least 1 hospital visit with a diagnosis of AF identified using the ICD-10 codes (I480-I484, I489) that occurred after enrollment in the study [25]. The index date was defined as the day of the first health examination between 2009 and 2012. Participants who did not have any events were censored at the time of death, last follow-up, or December 31, 2021, whichever came first.

## Covariates

The data collection method employed at the time of participant registration included a standardized questionnaire, which was completed by the participants themselves. This questionnaire collected information on smoking status (categorized as never- smoker, ex-smoker, or current smoker), alcohol consumption status , and physical activity level.Alcohol consumption status was classified as none, mild (<30 g/day for men, <20 g/day for women), moderate (≥30 g/day for men, ≥20 g/day for women), or excessive (≥60 g/day for men, ≥50 g/day for women). The detailed methodology for alcohol consumption assessment is provided in the Supplementary Methods [26]. Physical activity was assessed on the basis of engagement in moderate exercise for at least 30 min five or more days per week or intense exercise for at least 20 min three or more days per week. Low income status was defined as the lowest quantile based on income-related insurance contributions.

Diseases were identified via ICD-10 diagnostic codes, prescription records from the previous year, and health screening results. Hypertension was defined as the presence of diagnostic codes I10-13 or I15 along with a prescription for antihypertensive drugs or a systolic/diastolic blood pressure of 140/90 mmHg or higher. T2DM was identified by the use of diagnostic codes E11-14 and the presence of prescriptions for oral diabetic medications, insulin or a fasting glucose level of 126 mg/dL or higher. Dyslipidemia was indicated by the diagnostic code E78 and either the use of Lipid-lowering medications or a total Cholesterol level of 240 mg/dL or higher. Liver cirrhosis was defined using the ICD-10 codes K703 and K746, with associated one or more diagnoses during hospitalization or two or more diagnoses in outpatient clinics [27]. Ischemic heart disease was identified using ICD-10 codes I20-25 with associated hospitalization. Ischaemic stroke was defined as a diagnosis during admission using ICD-10 codes I63-64, with at least one claim for brain imaging studies, including magnetic resonance imaging/angiography or computed tomography [28]. Congestive heart failure was defined as hospitalization with the ICD-10 code I50 [29]. The estimated glomerular filtration rate (eGFR) was calculated from the serum creatinine levels via a formula derived from the Modification of Diet in Renal Disease Study [30].

### Statistical analysis

Prior to analysis, statistical outliers were identified and removed. Subsequently, we performed a complete case analysis, including only participants without missing data for the variables used in the models. Baseline clinical and demographic characteristics are presented as frequencies (%) for categorical variables and as the means ± standard deviations for continuous variables. Categorical variables were compared via Pearson’s χ^2^ test or Fisher’s exact test, whereas continuous variables were compared via Student’s *t*-test or the Mann–Whitney *U* test.

Incidence rates were calculated by dividing the number of events by the total follow-up period and are presented as rates per 1,000 person-years. Hazard ratios (HRs) and 95% confidence intervals (CIs) for the incidence of AF associated with MASLD, MetALD, and ALD were estimated using Cox proportional hazards models. Tests based on Schoenfeld residuals verified the proportional hazards assumption. We identified potential prognostic factors in advance, selected them on the basis of their clinical importance and performed an extensive review of the literature. The included factors were age, sex, low income, BMI, smoking status, regular physical activity, total cholesterol levels, fasting glucose levels, systemic blood pressure, and the eGFR. Sex, income, smoking, and regular exercise were modeled as categorical variables, whereas all other covariates were treated as continuous variables.

In addition, we conducted three sensitivity analyses. First, we adopted a higher cut-off of FLI ≥ 60 to define the presence SLD. Second, we assessed incident AF risk after excluding participants with AF-related comorbidities including valvular AF (defined using ICD-10 codes I05.0, I05.2, I05.9, Z95.2-Z95.4) and cardiomyopathy (defined using ICD‐10 code I42). Third, considering the recent changes in the nomenclatures of SLD, we compared AF incidence among NAFLD, metabolic dysfunction-associated fatty liver disease (MAFLD), and MASLD. All the statistical analyses were performed via SAS version 9.4 (SAS Institute, Cary, NC, USA). *P* values less than 0.05 indicated statistically significant differences.

## Results

### Study population

**Fig. 2:**
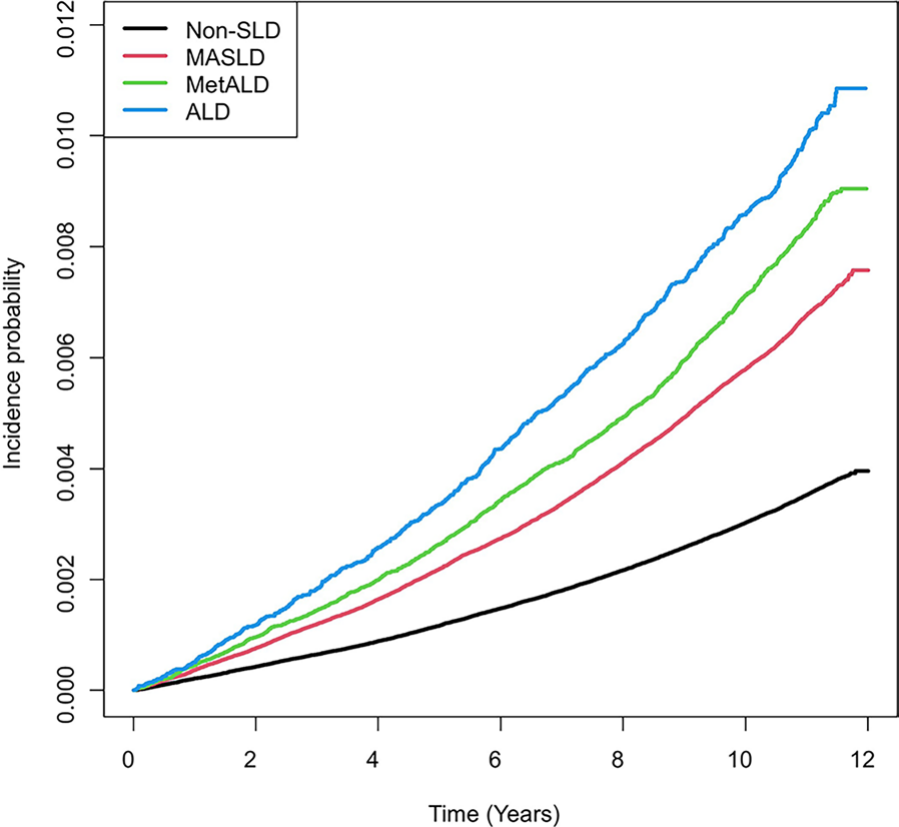
Probability of incident atrial fibrillation.This figure presents the Kaplan-Meier curves showing the incidence probability of AF over time. AF, atrial fibrillation; ALD, alcohol-associated liver disease; MASLD, metabolic dysfunction-associated steatotic liver disease; MetALD, metabolic dysfunction and alcohol- related liver disease; SLD, steatotic liver disease

A total of 6,375,710 subjects were included in this study, with a median follow-up duration of 10.6 years (interquartile range [IQR], 9.5–11.2) (Fig. 2). The mean age of the study population was 30.9 years, and the proportion of males was 59.2%. The prevalence of SLD was 27.8%, of which MASLD, MetALD, and ALD accounted for 81.7%, 13.5%, and 4.7%, respectively. The detailed baseline characteristics of the study population are presented in Table 1. Compared with the non-SLD subgroup, the proportion of males was greater in all SLD subgroups. In the MASLD, MetALD, and ALD groups, the proportion of individuals with a BMI greater than 23 exceeded 90%, and WC significantly increased. The prevalence of underlying cardiovascular disease, along with other comorbidities such as T2DM, hypertension, and dyslipidemia, was greater in the SLD group than in the non-SLD group. Most anthropometric and laboratory variables (including BMI, systolic/diastolic blood pressure, fasting glucose, total cholesterol, HDL and LDL cholesterol, and triglycerides) were more metabolically unfavorable in the SLD subgroups than in the non-SLD group.

**Table 1:** Baseline characteristics

Variables	Non-SLD(n=4,603,095)	SLD (n=1,783,384)			*P*-value
		MASLD (n=1,448,817)	MetALD (n=239,830)	ALD (n=83,968)	
Age, years	30.2 ± 5.0	32.7 ± 4.4	32.5 ± 4.4	32.5 ± 4.5	<0.001
Sex, N (%)					<0.001
Male	2,166,517 (47.1%)	1,310,519 (90.5%)	228,738 (95.4%)	80,590 (96.0%)	
Female	2,436,578 (52.9%)	138,298 (9.6%)	11,092 (4.6%)	3,378 (4.0%)	
BMI (kg/m^2^), N (%)	21.5 ± 2.5	27.0 ± 3.1	26.6 ± 3.1	26.8 ± 3.3	<0.001
Waist circumference (cm), N (%)	73.3 ± 7.3	88.5 ± 7.3	88.1 ± 7.3	88.6 ± 7.8	<0.001
Smoking, N (%)					<0.001
Never-smoker	2,970,687 (64.5%)	482,182 (33.3%)	31,753 (13.2%)	11,698 (13.9%)	
Ex-smoker	393,360 (8.6%)	210,183 (14.5%)	38,782 (16.2%)	13,259 (15.8%)	
Current smoker	1,239,048 (26.9%)	756,452 (52.2%)	169,315 (70.6%)	59,011 (70.3%)	
Drinking, N (%)					<0.001
Never	1,964,032 (42.7%)	429,682 (29.7%)	-	4,221 (5.0%)	
Mild	2,314,983 (50.3%)	1,019,135 (70.3%)	-	13,457 (16.0%)	
Moderate	275,912 (6.0%)	-	239,850 (100.0%)	4,819 (5.7%)	
Excessive	48,168 (1.0%)	-	-	61,471 (73.2%)	
Regular exercise, N (%)	573,124 (12.5%)	196,179 (13.5%)	35,123 (14.6%)	13,606 (16.2%)	<0.001
Low income, N (%)	1,078,212 (23.42)	233,196 (16.1)	34,939 (14.57)	13,691 (16.31)	<0.001
T2DM, N (%)	40,926 (0.9%)	64,473 (4.5%)	11,054 (4.6%)	5,275 (6.3%)	<0.001
Hypertension, N (%)	162,716 (3.5%)	242,434 (16.7%)	50,523 (21.1%)	20,189 (24.0%)	<0.001
Dyslipidemia, N (%)	145,533 (3.2%)	232,585 (16.1%)	37,227 (15.5%)	16,092 (19.2%)	<0.001
CKD, N (%)	105,734 (2.3%)	33,189 (2.3%)	5,141 (2.1%)	1,735 (2.1%)	<0.001
Liver cirrhosis, N (%)	416 (0.01)	224 (0.02)	17 (0.01)	138 (0.16)	<0.001
Cardiovascular disease, N (%)					
Ischemic heart disease	17,206 (0.37)	9,787 (0.68)	1,574 (0.66)	1,134 (1.35)	<0.001
Ischemic stroke	2,064 (0.04)	1,271 (0.09)	122 (0.05)	100 (0.12)	<0.001
Congestive heart failure	1,915 (0.04)	1,007 (0.07)	117 (0.05)	105 (0.13)	<0.001
Medication, N (%)					
Beta-blockers	11057 (0.24)	13176 (0.91)	2053 (0.86)	1367 (1.63)	<0.001
Calcium channel blockers	11324 (0.25)	21975 (1.52)	3778 (1.58)	2679 (3.19)	<0.001
Systolic blood pressure (mmHg)	114.7 ± 12.0	125.2 ± 12.8	127.3 ± 13.1	127.7 ± 13.2	<0.001
Diastolic blood pressure (mmHg)	71.8 ± 8.6	78.7 ± 9.4	80.4 ± 9.7	80.6 ± 9.8	<0.001
Fasting glucose (mg/dL)	88.8 ± 12.8	96.0 ± 22.7	97.2 ± 22.8	98.5 ± 25.8	<0.001
Total cholesterol (mg/dL)	177.3 ± 30.1	203.5 ± 35.6	202.9 ± 35.7	202.9 ± 36.8	<0.001
Triglyceride (mg/dL)	76.7 (76.6–76.7)	175.0 (174.8–175.1)	185.5 (185.1–185.9)	190.1 (189.4–190.8)	<0.001
HDL (mg/dL)	60.0 ± 21.9	49.8 ± 24.1	53.1 ± 20.4	53.3 ± 21.4	<0.001
LDL (mg/dL)	100.8 ± 31.2	116.0 ± 39.9	108.7 ± 39.4	107.2 ± 41.1	<0.001
eGFR (mL/min/1.73m^2^)	102.7 ± 21.7	99.1 ± 21.1	100.0 ± 20.8	100.4 ± 20.5	<0.001
AST (IU/L)	19.59 (19.59–19.60)	26.71 (26.70–26.73)	28.60 (28.56–28.65)	30.31 (30.22–30.39)	<0.001
ALT (IU/L)	15.62 (15.62–15.63)	34.16 (34.13–34.20)	34.20 (34.13–34.28)	36.64 (36.50–36.79)	<0.001
GGT (IU/L)	17.60 (17.59–17.60)	44.82 (44.78–44.87)	64.54 (64.38–64.71)	70.95 (70.62–71.28)	<0.001

ALD, alcohol-associated liver disease; ALT, alanine aminotransferase; AST, aspartate aminotransferase; CKD, chronic kidney disease; eGFR, estimated glomerular filtration rate; GGT, gamma-glutamyl transferase; HDL, high-density lipoprotein; LDL, low-density lipoprotein; MASLD, metabolic dysfunction-associated steatotic liver disease; MetALD, metabolic dysfunction and alcohol-related liver disease; SLD, steatotic liver disease; T2DM, type 2 diabetes mellitus

## Risk of new-onset AF in young adults with SLD

We compared the probability of AF over time by SLD subtype. Figure 2 shows the Kaplan-Meier curves of the incidence probability of AF over time. The incidence of AF progressively increased in the order of MASLD, MetALD, and ALD compared to the non-SLD group (log-rank test, *P* < 0.001). After adjustment for potential confounders, including age, sex, income, smoking status, regular physical activity, total cholesterol level, fasting blood glucose level, systolic blood pressure, estimated glomerular filtration rate, and BMI (Model 2), the presence of any SLD was associated with a 14% increased risk of incident AF (adjusted HR [aHR]: 1.14, 95% CI: 1.10–1.18) compared with the non-SLD group. When the analysis was stratified by SLD subtype, all subtypes remained significantly associated with a higher risk of AF after full adjustment. The risk was most pronounced in young adults with ALD (aHR: 1.52, 95% CI: 1.41–1.65). An elevated risk was also observed in those with MetALD (aHR: 1.29, 95% CI: 1.22–1.36) and MASLD (aHR: 1.09, 95% CI: 1.05–1.31), all compared to the non-SLD group (Table 2). This trend persisted after adjusting for alcohol consumption (10 g/day) as a continuous variable (Supplementary Table 2). To evaluate the effect of cardiometabolic risk factors (CMRFs) in the context of ALD, we compared the HRs for AF in ALD patients stratified by the presence of CMRFs. The HR was greater in those without CMRFs than in those with CMRFs (1.99 vs. 1.52, Supplementary Table 3); however, the difference was not statistically significant because of the small number of events in the ALD without CMRFs group (*n* = 6).

**Table 2:** Risk of incident atrial fibrillation in young adults with SLD

Subgroup	Number	Number of events	Follow-up duration (*p*-y)	Incidence rate (per 1,000 *p*-y)	Crude HR (95% CI)	Adjusted HR (95% CI)	
						Model 1*	Model 2*
**Non-SLD**	4,603,095	14,772	47,427,013	0.31	1.00 (Reference)	1.00 (Reference)	1.00 (Reference)
**All SLD**	1,772,615	11,620	18,387,460	0.63	2.02 (1.97 –2.07)	1.49 (1.45 –1.53)	1.14 (1.10– 1.18)
**MASLD**	1,448,817	9,014	15,032,918	0.60	1.92 (1.87–1.97)	1.41 (1.37–1.45)	1.09 (1.05–1.31)
**MetALD**	239,830	1,834	2,486,934	0.74	2.36 (2.24–2.47)	1.73 (1.64–1.81)	1.29 (1.22–1.36)
**ALD**	83,968	772	867,609	0.89	2.85 (2.65–3.06)	2.08 (1.93–2.24)	1.52 (1.41–1.65)

* Model 1 adjusted by age and sex; Model 2 adjusted by age, sex, low income status, smoking, regular exercise, total cholesterol, fasting blood glucose level, systolic blood pressure, estimated glomerular filtration rate, and body mass index

ALD, alcohol-associated liver disease; CI, confidence interval; ; HR, hazard ratio; MASLD, metabolic dysfunction-associated steatotic liver disease; MetALD, metabolic dysfunction and alcohol-related liver disease; p-y, person-years; SLD, steatotic liver disease

### Subgroup analysis of the risk of AF in young adults with SLD

We performed subgroup analyses stratified by age, sex, obesity status (BMI < 25 kg/m² vs. ≥25 kg/m²), and smoking status. The increased risk of incident AF across all SLD subtypes was more pronounced in individuals aged 30 years or older than in those under 30 years (*P* for interaction = 0.009; Table 3). Furthermore, obese individuals presented a greater relative risk of AF across the MASLD and MetALD subtypes than their nonobese counterparts did, whereas the elevated AF risk associated with ALD was more pronounced among nonobese individuals (*P* for interaction = 0.03). The increased AF risk in the MASLD and ALD subgroups compared with that in the non-SLD subgroup was consistent regardless of sex and smoking status.

**Table 3:** Stratified analysis of incident atrial fibrillation risk among young adults with SLD

Variables	Subgroups	Number of events	Follow-up duration (person-years)	Incidence rate (per 1,000 *p*-y)	Adjusted HR (95% CI)	*P* for interaction
Age <30 years	Non-SLD	5,069	22,737,650	0.22	1.00 (Reference)	0.001
	MASLD	1,520	3,814,040	0.40	1.05 (0.99– 1.12)	
	MetALD	314	698,612	0.45	1.14 (1.02– 1.28)	
	ALD	123	245,736	0.50	1.25 (1.04– 1.49)	
Age ≥30 years	Non-SLD	9,703	24,689,364	0.39	1.00 (Reference)	
	MASLD	7,494	11,218,878	0.67	1.10 (1.06– 1.14)	
	MetALD	1,520	1,788,323	0.85	1.32 (1.25– 1.41)	
	ALD	649	621,873	1.04	1.59 (1.47– 1.73)	
Male	Non-SLD	8,915	2,2450,770	0.40	1.00 (Reference)	0.782
	MASLD	8,391	13,630,884	0.62	1.09 (1.05– 1.13)	
	MetALD	1,786	2,375,902	0.75	1.29 (1.22– 1.37)	
	ALD	750	834,022	0.90	1.52 (1.40– 1.64)	
Female	Non-SLD	5,857	24,976,243	0.23	1.00 (Reference)	
	MASLD	623	1,402,034	0.44	1.10 (1.00– 1.20)	
	MetALD	48	111,032	0.43	1.14 (0.85– 1.52)	
	ALD	22	33,587	0.66	1.72 (1.13– 2.62)	
BMI <25 kg/m^2^	Non-SLD	13,126	43,328,918	0.30	1.00 (Reference)	0.033
	MASLD	2,052	4,034,079	0.51	1.05 (1.00– 1.10)	
	MetALD	474	785,150	0.60	1.20 (1.09– 1.32)	
	ALD	217	263,440	0.82	1.65 (1.44– 1.88)	
BMI ≥25 kg/m^2^	Non-SLD	1,646	4,098,096	0.40	1.00 (Reference)	
	MASLD	6,962	10,998,839	0.63	1.13 (1.07–1.20)	
	MetALD	1,360	1,701,785	0.80	1.35 (1.26– 1.46)	
	ALD	555	604,168	0.92	1.52 (1.38– 1.67)	
Never-smoker	Non-SLD	8,034	30,520,976	0.26	1.00 (Reference)	0.267
	MASLD	2,580	4,973,004	0.52	1.09 (1.03–1.15)	
	MetALD	185	326,978	0.57	1.16 (1.00–1.35)	
	ALD	95	121,004	0.79	1.53 (1.25– 1.88)	
Ex-smoker	Non-SLD	1,897	4,101,965	0.46	1.00 (Reference)	
	MASLD	1,594	2,202,697	0.72	1.06 (0.99– 1.13)	
	MetALD	347	405,983	0.85	1.23 (1.10– 1.38)	
	ALD	130	138,496	0.94	1.31 (1.10– 1.57)	
Current smoker	Non-SLD	4,841	12,804,072	0.38	1.00 (Reference)	
	MASLD	4,840	7,857,217	0.62	1.11 (1.06– 1.16)	
	MetALD	1,302	1,753,973	0.74	1.33 (1.25– 1.42)	
	ALD	547	608,109	0.90	1.59 (1.45– 1.74)	

* Adjusted by age, sex, low income status, smoking, regular exercise, total cholesterol, fasting blood glucose level, systolic blood pressure, estimated glomerular filtration rate, and body mass index

ALD, alcohol-associated liver disease; BMI, body mass index; CI, confidence interval; ; HR, hazard ratio; MASLD, metabolic dysfunction-associated steatotic liver disease; MetALD, metabolic dysfunction and alcohol-related liver disease; SLD, steatotic liver disease

### Sensitivity analysis

To evaluate the robustness of our findings, we conducted several sensitivity analyses. First, applying a higher FLI cutoff value (≥ 60) to define SLD yielded results similar to those of the primary analysis (Supplementary Table 4). Second, we excluded participants with AF-related comorbidities such as valvular AF and cardiomyopathy. The results were consistent with our primary analysis, confirming the robust association between SLD subtypes and incident AF (Supplementary Table 5**)**. Finally, given the evolving diagnostic nomenclature, we compared the incidence of AF across NAFLD, MAFLD, and MASLD definitions. The results revealed that the risk of incident AF consistently increased across all three definitions compared with that of participants without SLD (Supplementary Table 6).

### AF risk according to FLI change

To account for the dynamic nature of SLD, we stratified participants according to changes in SLD status over a 2-year follow-up period. Individuals with persistent SLD or newly developed SLD during follow-up presented a significantly greater risk of incident AF than those who remained consistently free of SLD (Supplementary Table 7). These results suggest that both the presence and progression of hepatic steatosis contribute to the risk of developing AF.

## Discussion

In this nationwide cohort study involving over six million young adults aged 20–39 years, the risk of incident AF was significantly greater in subjects with SLD than in those without SLD. Importantly, the magnitude of AF risk varied across SLD subtypes, with the adjusted HR being the highest in individuals with ALD, followed by those with MetALD and MASLD. These findings suggest that new-onset AF was positively associated with SLD in young adults and that the association was most pronounced in the ALD subgroup.

A previous study conducted within the same age group demonstrated an association between NAFLD and incident AF risk [17]. Our study extends this observation by applying the newly established SLD nomenclature, thereby providing SLD subtype-specific estimates of AF risk. This detailed classification provides insight into how metabolic dysfunction and alcohol exposure jointly influence AF risk. A recent large-scale meta-analysis has established that MASLD is associated with an increased long-term risk of developing AF [15]. However, most existing evidence is derived from middle-aged or older populations, in whom the prevalence of both MASLD and traditional AF risk factors such as hypertension, diabetes, and advanced age is highest. This leaves a critical knowledge gap regarding the nature of this association in younger individuals. Our study addresses this gap by focusing specifically on the association between MASLD and incident AF in a young adult population. By demonstrating this association, our study suggests that the pathophysiological mechanism underlying AF might begin much earlier than previously recognized. These results indicate that MASLD serves as a relevant risk factor not only in older adults but also in younger individuals.

The increasing prevalence of MASLD in young adults is largely driven by the increasing rates of severe obesity and the growing burden of cardiometabolic risk factors in overweight and obese children and young adults [31, 32]. In our study, young adults with MASLD presented a statistically significant increase in the risk of incident AF, with an aHR of 1.11. This risk was more prominent among individuals aged 30 years or older than among younger individuals, and obese individuals had a greater relative risk of AF in the MASLD and MetALD subtypes than their nonobese counterparts did. These findings suggest that age-related cumulative cardiometabolic burden and obesity increase the arrhythmogenic potential of MASLD. MASLD encompasses a cluster of independent risk factors for AF, including obesity, insulin resistance, hypertension, and systemic inflammation [33]. These risk factors contribute to both hepatic fat accumulation and atrial remodeling, thereby increasing susceptibility to AF [34]. The underlying pathophysiological mechanisms linking MASLD and AF are thought to involve chronic low-grade inflammation, oxidative stress, and mitochondrial dysfunction, all of which play critical roles in promoting changes to atrial structure and electrical activity that predispose patients to arrhythmia [35–37]. Individuals with hepatic steatosis exhibit elevated serum levels of systemic inflammatory markers [38], and MASLD further promotes oxidative stress through increased production of reactive oxygen species in hepatocytes [39]. In addition, increased epicardial adipose tissue (EAT) has been associated with local inflammation and atrial enlargement in MASLD [40]. Obesity amplifies these effects by increasing EAT, hemodynamic load, and systemic inflammation [41]. Further research is needed to clarify the mechanisms driving AF in young adults with SLD.

In our study, the adjusted HR of incident AF was greater in the MetALD and ALD groups than in the MASLD group, suggesting that a higher degree of alcohol consumption was associated with an increased risk of AF. These findings indicate that the interaction between hepatic steatosis and alcohol consumption likely contributes to increased AF risk in young adults. The process of alcohol metabolism in the liver promotes hepatic steatosis by enhancing fatty acid synthesis while inhibiting fatty acid oxidation [42]. In addition, alcohol-induced hepatic steatosis may trigger systemic inflammation and oxidative stress, which are known to play key roles in the development and maintenance of AF [43, 44]. Furthermore, alcohol itself can directly exert electrophysiological and autonomic effects, contributing to the development of AF [9]. Some pathophysiological mechanisms through which alcohol directly contributes to the development of AF have been identified. First, alcohol consumption may induce electrical atrial remodeling, producing an arrhythmogenic substrate [45–47]. Second, alcohol affects the sympathetic and parasympathetic nervous systems, which may contribute to AF [48, 49]. Third, alcohol has direct effects on atrial excitation‒contraction coupling and may contribute to tissue fibrosis, leading to structural remodeling of the atria [50, 51]. Therefore, in addition to systemic inflammation and oxidative stress, which are shared pathways with MASLD, alcohol may influence electrophysiological properties and cardiac structure through independent mechanisms. These effects may explain our findings that alcohol consumption was associated with an increased risk of incident AF among young adults with hepatic steatosis.

The male predominance among patients with ALD and MetALD is attributed primarily to well-documented gender disparities in alcohol consumption patterns [3, 52]. This established trend is further compounded in the case of MASLD, where the gender-specific epidemiology is significantly influenced by the earlier onset and higher prevalence of metabolic syndrome in men, particularly during young adulthood and middle age [53]. 

This study is the first to assess the lifetime risk of AF in young adults with SLD within an Asian population, utilizing comprehensive data from the South Korean NHIS. Given that the universal health insurance system of South Korea covers nearly the entire population, the use of this dataset ensures reliable longitudinal data and minimizes the risk of missing information, thereby increasing the robustness of the analysis [54].

However, our study also has limitations. First, although histological and radiological assessments are the gold standard, their feasibility in large-scale epidemiological studies is limited. Therefore, the FLI was utilized to address these limitations [19, 20]. Second, the observational nature of this population-based study precludes causal inference, and residual confounding cannot be excluded. Additionally, the use of claims data on the basis of ICD-10 codes introduces the potential for misclassification bias.

In conclusion, young adults with SLD have a significantly elevated risk of new-onset AF, with progressive risk increases across MASLD, MetALD, and ALD subtypes. Given the modifiable nature of these risks, identification and management of SLD, particularly in those who consume alcohol, may be essential for reducing the future burden of AF and its associated cardiovascular complications.

## Supplementary Information

Supplementary Material 1

## Data Availability

The dataset (NHIS-HEALS) supporting the conclusions of this article is available in the homepage of National Health Insurance Sharing Service [http://nhiss.nhis.or.kr/bd/ab/bdaba021eng.do]. To gain access to the data, a completed application form, a research proposal and the applicant’s approval document from the institutional review board should be submitted to and reviewed by the inquiry committee of research support in NHIS. Currently, use of NHIS data is allowed only for Korean researchers.

## Abbreviations

AF: Atrial fibrillation
ALD: Alcohol-associated liver disease
BMI: Body mass index
CI: Confidence interval
CLD: Chronic liver disease
CVD: Cardiovascular disease
FLI: Fatty liver index
GGT: Gamma-glutamyl transferase
HR: Hazard ratio
ICD-10: International classification of diseases, 10th revision
IQR: Interquartile range
NAFLD: nonalcoholic fatty liver disease
MASLD: Metabolic dysfunction-associated steatotic liver disease
MetALD: Metabolic dysfunction and alcohol-related liver disease
NHIS: National Health Insurance Service
SLD: Steatotic liver disease
T2DM: Type 2 diabetes mellitus
WC: Waist circumference
