# Supplementary material for: Associations between steatotic liver disease subtypes and incident atrial fibrillation in young adults: a nationwide cohort study
Source: Cardiovasc Diabetol. 2025 Aug 25;24:348. doi: 10.1186/s12933-025-02905-3 (PMC12376484; doi:10.1186/s12933-025-02905-3)
Supplement: Supplementary file 1 — Supplementary Material 1 [file 12933_2025_2905_MOESM1_ESM.docx]

**Supplementary Methods**. The questionnaire regarding alcohol consumption in 2009 Korean nationwide health check-up (translated into English).

| Please read the following questions and fill out your current situation.  - On average, how many days a week do you drink?  □ 0 □ 1 □ 2 □ 3 □ 4 □ 5 □ 6 □ 7  - How much do you usually drink a day when you drink? (Cup)  (※ We calculate with glass used for each liquor type. One can of beer (355cc) is equivalent to 1.6 cups of beer) |
| --- |

**Supplement Table 1. Diagnostic codes used in this study.**

| Conditions | Definition |
| --- | --- |
| Viral hepatitis (e.g. hepatitis B, C) | ICD 10: B15-19, B00.8, B25.1 |
| Hepatic veno-occlusive disease | ICD 10: I82 |
| Drug-induced liver disease | ICD 10: K71 |
| Autoimmune hepatitis | ICD 10: K75.4 |
| Primary biliary cholangitis | ICD 10: K74.3, K74.4 |
| Liver abscess | ICD 10: K75.0, A 06.4 |
| Wilson’s disease | ICD 10: E83.0 |
| Hemochromatosis | ICD 10: E83.1 |
| Alpha-1 antitrypsin deficiency | ICD 10: E88.0 |
| Other cholangitis | ICD 10: K83, K83.0 |
| Glycogen storage disease | ICD 10: E74 |
| Alcohol abuse/ alcohol-related liver disease | ICD 10: E24.4, F10, G31.2, G62.1, G72.1, I42.6, K29.2, K70, K86.0, Q35.4, R78.0, T51.0, T51.8, T51.9, X65, Y15, Y57.3, Y90, Y91, Z50.2, Z71.4 |
| Hypertension | ICD 10: I10-13 or I15 |
| Type 2 diabetes mellitus | ICD 10: E11-14 |
| Dyslipidemia | ICD 10: E78 |
| Liver cirrhosis | ICD 10: K703 and K746 |
| Ischemic heart disease | ICD 10: I20–25 with associated hospitalization |
| Ischemic stroke | ICD 10: I63–64 during admission with at least one claim for brain imaging studies |
| Congestive heart failure | ICD-10 code I50 with hospitalization |
| AF | I480-I484, I489 |
| valvular AF | ICD‐10 codes I05.0, I05.2, I05.9, Z95.2-Z95.4 |
| cardiomyopathy | ICD‐10 codes I42 |

ICD, International Classification of Diseases

**Supplementary Table 2. Risk of incident atrial fibrillation in young adults with SLD**

| **Subgroup** | **Number** | **Number of events** | **Follow-up duration**  **(p-y)** | **Incidence rate**  **(per 1,000 p-y)** | **Adjusted HR (95% CI)** | | |
| --- | --- | --- | --- | --- | --- | --- | --- |
|  |  |  |  |  | **Model 1*** | **Model 2*** | **Model 3*** |
| **Non-SLD** | 4,603,095 | 14,772 | 47,427,013 | 0.31 | 1.00 (Reference) | 1.00 (Reference) | 1.00 (Reference) |
| **MASLD** | 1,448,817 | 9,014 | 15,032,918 | 0.60 | 1.41 (1.37–1.45) | 1.09 (1.05–1.31) | 1.11 (1.07–1.15) |
| **MetALD** | 239,830 | 1,834 | 2,486,934 | 0.74 | 1.73 (1.64–1.81) | 1.29 (1.22–1.36) | 1.15 (1.06–1.24) |
| **ALD** | 83,968 | 772 | 867,609 | 0.89 | 2.08 (1.93–2.24) | 1.52 (1.41–1.65) | 1.49 (1.34–1.66) |

* Model 1 adjusted by age and sex; Model 2: adjusted by age, sex, low income status, smoking, regular exercise, total cholesterol, fasting blood glucose level, systolic blood pressure, estimated glomerular filtration rate, and body mass index; Model 3: In addition to model 1, further adjusted for alcohol consumption as a continuous variable.

Abbreviation: ALD, alcohol-associated liver disease; CI, confidence interval; HR, hazard ratio; MASLD, metabolic dysfunction-associated steatotic liver disease; MetALD, metabolic and alcohol-associated liver disease; p-y, person-years; SLD, steatotic liver disease.

**Supplementary Table 3.** Risk of incident atrial fibrillation in young adults with ALD according to the presence or absence of CMRF.

| **Subgroups** | **Number of events** | **Follow-up duration**  **(p-y)** | **Incidence rate**  **(per 1,000 p-y)** | **Crude HR (95% CI)** | **Adjusted HR (95% CI)** | |
| --- | --- | --- | --- | --- | --- | --- |
|  |  |  |  |  | **Model 1*** | **Model 2*** |
| **Non-SLD** | 14,772 | 47,427,013 | 0.31 | 1.00 (Reference) | 1.00 (Reference) | 1.00 (Reference) |
| **MASLD** | 9,014 | 15,032,918 | 0.60 | 1.92 (1.87–1.97) | 1.41 (1.37–1.45) | 1.09 (1.05–1.31) |
| **MetALD** | 1,834 | 2,486,934 | 0.74 | 2.36 (2.24–2.47) | 1.73 (1.64–1.81) | 1.29 (1.22–1.36) |
| **ALD without CMRF** | 6 | 7,341 | 0.82 | 2.61 (1.17–5.81) | 1.93 (0.87–4.30) | 1.99 (0.90–4.44) |
| **ALD with CMRF** | 766 | 860,268 | 0.89 | 2.85 (2.65–3.06) | 2.08 (1.93–2.24) | 1.52 (1.41–1.64) |

* Model 1 adjusted by age and sex; Model 2 adjusted by age, sex, low income status, smoking, regular exercise, total cholesterol, fasting blood glucose level, systolic blood pressure, estimated glomerular filtration rate, and body mass index.

Abbreviation: ALD, alcohol-associated liver disease; CI, confidence interval; CMRF, cardio-metabolic risk factor; HR, hazard ratio; MASLD, metabolic dysfunction-associated steatotic liver disease; MetALD, metabolic and alcohol-associated liver disease; p-y, person-years; SLD, steatotic liver disease.

**Supplementary Table 4.** Risk of incident atrial fibrillation in young adults with SLD using fatty liver index cutoffs of 60.

| **Subgroups** | **Number** | **Number of events** | **Follow-up duration**  **(p-y)** | **Incidence rate**  **(per 1,000 p-y)** | **Crude HR (95% CI)** | **Adjusted HR (95% CI)** | |
| --- | --- | --- | --- | --- | --- | --- | --- |
|  |  |  |  |  |  | **Model 1*** | **Model 2*** |
| **Non-SLD** | 5,641,004 | 20,610 | 58,214,403 | 0.35 | 1.00 (Reference) | 1.00 (Reference) | 1.00 (Reference) |
| **MASLD** | 570,398 | 4,270 | 5,900,631 | 0.72 | 2.04 (1.97–2.11) | 1.53 (1.48–1.59) | 1.14 (1.09–1.18) |
| **MetALD** | 118,616 | 1,057 | 1,227,694 | 0.86 | 2.42 (2.28–2.59 | 1.80 (1.69–1.91) | 1.32 (1.23–1.41) |
| **ALD** | 45,692 | 455 | 471,748 | 0.96 | 2.72 (2.48–2.98) | 2.02(1.84–2.21) | 1.45 (1.32–1.60) |

* Model 1 adjusted by age and sex; Model 2 adjusted by age, sex, low income status, smoking, regular exercise, total cholesterol, fasting blood glucose level, systolic blood pressure, estimated glomerular filtration rate, and body mass index.

Abbreviation: ALD, alcohol-associated liver disease; CI, confidence interval; HR, hazard ratio; MASLD, metabolic dysfunction-associated steatotic liver disease; MetALD, metabolic and alcohol-associated liver disease; p-y, person-years; SLD, steatotic liver disease.

**Supplementary Table 5.** Risk of incident atrial fibrillation in young adults with SLD after excluding those with valvular heart disease or cardiomyopathy

| **Subgroups** | **Number** | **Number of events** | **Follow-up duration**  **(p-y)** | **Incidence rate**  **(per 1,000 p-y)** | **Crude HR (95% CI)** | **Adjusted HR (95% CI)** | |
| --- | --- | --- | --- | --- | --- | --- | --- |
|  |  |  |  |  |  | **Model 1*** | **Model 2*** |
| **Non-SLD** | 4,600,832 | 14,240 | 47,406,147 | 0.30 | 1.00 (Reference) | 1.00 (Reference) | 1.00 (Reference) |
| **MASLD** | 1,447,869 | 8,655 | 15,024,855 | 0.58 | 1.91 (1.86–1.96) | 1.41 (1.37–1.45) | 1.09 (1.05–1.13) |
| **MetALD** | 239,696 | 1,765 | 2,485,850 | 0.71 | 2.35 (2.24–2.47) | 1.72 (1.64–1.81) | 1.29 (1.22–1.36) |
| **ALD** | 83,910 | 745 | 867,103 | 0.86 | 2.85 (2.65–3.07) | 2.08 (1.93–2.24) | 1.53 (1.41–1.66) |

* Model 1 adjusted by age and sex; Model 2 adjusted by age, sex, low income status, smoking, regular exercise, total cholesterol, fasting blood glucose level, systolic blood pressure, estimated glomerular filtration rate, and body mass index.

Abbreviation: ALD, alcohol-associated liver disease; CI, confidence interval; HR, hazard ratio; MASLD, metabolic dysfunction-associated steatotic liver disease; MetALD, metabolic and alcohol-associated liver disease; p-y, person-years; SLD, steatotic liver disease.

**Supplementary Table 6.** Incidence of atrial fibrillation among NAFLD/MAFLD/MASLD patients.

| **Subgroups** | **Number** | **Number of events** | **Follow-up duration**  **(person-years)** | **Incidence rate**  **(per 1,000 p-y)** | **Crude HR (95% CI)** | **Adjusted HR (95% CI)** | |
| --- | --- | --- | --- | --- | --- | --- | --- |
|  |  |  |  |  |  | **Model 1*** | **Model 2*** |
| **No steatosis** | 4,603,095 | 14,772 | 47,427,013 | 0.31 | 1.00 (Reference) | 1.00 (Reference) | 1.00 (Reference) |
| **NAFLD** | 1,474,024 | 9,239 | 15,295,586 | 0.60 | 1.93 (1.88–1.98) | 1.42 (1.38–1.46) | 1.10 (1.06–1.14) |
| **No steatosis** | 4,603,095 | 14,772 | 47,427,013 | 0.31 | 1.00 (Reference) | 1.00 (Reference) | 1.00 (Reference) |
| **MAFLD** | 1,727,002 | 11,375 | 17,911,576 | 0.64 | 2.03 (1.98–2.08) | 1.49 (1.45–1.53) | 1.13 (1.09–1.17) |
| **No steatosis** | 4,603,095 | 14,772 | 47,427,013 | 0.31 | 1.00 (Reference) | 1.00 (Reference) | 1.00 (Reference) |
| **MASLD** | 1,448,817 | 9,014 | 15,032,918 | 0.60 | 1.92 (1.87–1.97) | 1.41 (1.37–1.45) | 1.09 (1.05–1.31) |
| **MetALD** | 239,830 | 1,834 | 2,486,934 | 0.74 | 2.36 (2.24–2.47) | 1.73 (1.64–1.81) | 1.29 (1.22–1.36) |
| **ALD** | 83,968 | 772 | 867,609 | 0.89 | 2.85 (2.65–3.06) | 2.08 (1.93–2.24) | 1.52 (1.41–1.65) |

* Model 1 adjusted by age and sex; Model 2 adjusted by age, sex, low income status, smoking, regular exercise, total cholesterol, fasting blood glucose level, systolic blood pressure, estimated glomerular filtration rate, and body mass index.

Abbreviation: ALD, alcohol-associated liver disease; CI, confidence interval; HR, hazard ratio; NAFLD, nonalcoholic fatty liver disease; MAFLD, metabolic dysfunction-associated fatty liver disease; MASLD, metabolic dysfunction-associated steatotic liver disease; MetALD, metabolic and alcohol-associated liver disease; SLD, steatotic liver disease. and alcohol-associated liver disease; SLD, steatotic liver disease.

**Supplementary Table 7.** Risk of incident atrial fibrillation according to the changes of steatosis.

| **Initial** | **Follow up**  **FLI** | **Number** | **Number of events** | **Incidence rate**  **(per 1,000 p-y)** | **Crude HR (95% CI)** | **Adjusted HR (95% CI)** | |
| --- | --- | --- | --- | --- | --- | --- | --- |
|  |  |  |  |  |  | **Model 1*** | **Model 2*** |
| **Non-SLD** | < 30 | 2,377,277 | 7,003 | 0.31 | 1.00 (Reference) | 1.00 (Reference) | 1.00 (Reference) |
|  | 30-59 | 285,889 | 1,321 | 0.49 | 1.56 (1.47–1.65) | 1.26 (1.18–1.34) | 1.13 (1.06–1.21) |
|  | ≥ 60 | 37,584 | 199 | 0.57 | 1.82 (1.58–2.10) | 1.56 (1.35–1.79) | 1.31 (1.13–1.52) |
| **MASLD** | < 30 | 151,270 | 721 | 0.50 | 1.59 (1.47–1.71) | 1.19 (1.10–1.29) | 1.08 (1.00–1.17) |
|  | 30-59 | 362,822 | 2,030 | 0.59 | 1.86 (1.77–1.95) | 1.33 (1.26–1.40) | 1.15 (1.08–1.22) |
|  | ≥ 60 | 395,220 | 2,701 | 0.72 | 2.30 (2.20–2.40) | 1.66 (1.59–1.74) | 1.29 (1.20–1.38) |
| **MetALD** | < 30 | 17,628 | 94 | 0.56 | 1.78 (1.45–2.18) | 1.32 (1.08–1.62) | 1.19 (0.97–1.46) |
|  | 30-59 | 51,333 | 343 | 0.70 | 2.22 (1.99–2.47) | 1.59 (1.43–1.78) | 1.37 (1.22–1.53) |
|  | ≥ 60 | 79,146 | 669 | 0.89 | 2.83 (2.62–3.07) | 2.04 (1.88–2.21) | 1.58 (1.44–1.73) |
| **ALD** | < 30 | 5,350 | 33 | 0.65 | 2.06 (1.46–2.89) | 1.53 (1.09–2.16) | 1.38 (0.98–1.94) |
|  | 30-59 | 15,558 | 127 | 0.86 | 2.71 (2.28–3.23) | 1.93 (1.60–2.30) | 1.65 (1.38–1.98) |
|  | ≥ 60 | 28,504 | 255 | 0.94 | 3.00 (2.65–3.40) | 2.14 (1.89–2.43) | 1.13 (1.06–1.21) |

* Model 1 adjusted by age and sex; Model 2 adjusted by age, sex, low income status, smoking, regular exercise, total cholesterol, fasting blood glucose level, systolic blood pressure, estimated glomerular filtration rate, and body mass index.

Abbreviation: ALD, alcohol-associated liver disease; CI, confidence interval; CVD, cardiovascular disease; FLI, fatty liver index; HR, hazard ratio; MASLD, metabolic dysfunction-associated steatotic liver disease; MetALD, metabolic and alcohol-associated liver disease; SLD, steatotic liver disease.
